# Selections and Abstracts

**Published:** 1905-05

**Authors:** 


### SELECTIONS AND ABSTRACTS.

### A Case-History Clinic.*

Richard C. Cabot, M.D., Boston, Mass.

(The following stenographic notes are a record of a demonstration by the author of a method of clinical instruction used by himself at the Harvard Medical School. No patient was present. Typewritten slips containing the case-history as given below were distributed among the students. It was taken for granted that the physical examination had been made, clinical and laboratory findings, and the history all known. With these at hand, the class is led to deduce the diagnosis, prognosis and treatment. The great interest of this method and its value to the class is shown by the following report. The notes on one case have been deemed sufficient.—S., here means student.—Editor.)

"A factory overseer, sixty-three years old, had been subject to constipation for five years, and for two years had had right inguinal hernia. Otherwise his antecedents from a medical point of view were excellent. On the day before his illness he had what he regarded as a satisfactory movement of the bowels. That night he ate heartily of clam-chowder and strawberries. The next afternoon he felt some abdominal discomfort. Later, while taking a bath, he found his hernia was down (as he had taken the truss off), and he found more difficulty than usual in replacing it. That night he vomited a great many times, the first vomitus suggesting strawberries, and he had great abdominal pain, diffuse, not localized. At 4 the next morning, when seen for the first time, he was not collapsed. The tongue was moist, with a slight white coat. Temp. 98.4, pulse 60, respiration 14. The abdomen was soft, not tender. The hernia was found to be perfectly reduced. Nothing abnormal was felt per anum. The pain required an injection of ½ grain morphia. Nausea was so troublesome that the patient refused even bits of ice. (Nothing whatever passed the bowels.) On the second day the vomiting became stercoraceous. On the third day the vomiting persisted. Temp. 98.5, pulse 68. Large

*Given before Dr. Dock's clinic, University Hospital, Ann Arbor, March 21, 1905.

enemata (5½ quarts) had been given three times without apparent benefit. The belly was distended, rather hard, not tender. In the right side an ill-defined resistance seemed to be felt, corresponding to the ascending colon. A consultation was now called. What would have been your diagnosis, prognosis, and treatment?"

Mr. Barnett. You note that the respiration is 14. What comes to your mind as a possible cause for that respiration? The best way when confronted with a fact of this kind is to think what are the causes of slow respiration anyway, and then go through the different causes and see if any of them will fit this case. We know of a great many things which cause rapid respiration, but unfortunately, I asked for those of slow respiration, a question much less easy to answer. S.—Toxemia. Dr.—What are the effects, of toxemia for instance, in typhoid and uremia? S.—The first effect is to increase the respiration. Dr.—Yes, that is the usual effect; what other? S.—It starts with a slow respiration and works up to a climax, and then goes down again. Dr.—Then it is a rhythmical increase and decrease of the rate and depth of respiration, followed by a period of apnea, i. e., when there is no breathing at all. What are some of the causes of that? S.—You get it in a good many acute respiratory disorders, such as pleurisy. Dr.—Not often in pleurisy, but in what other acute infection? S.—Pneumonia. Dr.—Yes, and that is one of the times when it does not mean death. You think of it chiefly as a prognostic sign; you see it and you know death is near, but in pneumonia you see it, and yet the patient may get well. S.—You see it in nervous disorders. Dr.—I think it is mostly in brain tumor, meningitis and organic brain diseases of that kind. Mr. Bower, can you tell me of any condition in which a patient may have Cheyne-Stokes and yet be normal? When does it occur in health? S.—It occurs in young children when they are asleep. Dr.—Yes, if you watch you will see Cheyne-Stokes under those conditions. It is commoner than we often suppose; we ordinarily look for it only when people are sick. But we are far afield from our original point. Why has this patient got slow breathing? S.—If he has much abdominal pain, the breathing might be slow. Dr.—Well, it might be restrained, but would it necessarily be slow? In peritonitis, for example, do they have slow breathing? No; shallow and of a costal type. Is there any possible cause of that respiration 14? S.—I don't know of any other causes. Dr.—Well, we have not mentioned any yet. S.—Abdominal tenderness. Dr.—I don't think that is a cause of slow breathing. It is a cause of shallow breathing or costal breathing, but not slow breathing. S.—The time of day would have something to do with it. Dr.—What time is it slowest? S.—Early morning. Dr.—What time of day is it quickest? S.—In the evening. Dr.—It is slow in the early morning hours, and quickens as the day goes through. Any other cause? S.—The slow pulse-rate. Dr.—I don't think I can agree with that. One has a great many cases of slow pulse-rate that don't produce slow breathing. S.—You get slow breathing from poisonous vapor. Dr.—You get it occasionally in gas or shock. The question is, whether this case is due to shock. S.—Some drug, perhaps. Dr.—What drug could produce slow breathing? S.—Morphine. Dr.—How do you know he hasn't had morphine? The first question you should ask yourself is, has he had morphine? I brought that point up because if you were confronted with a case in that way, the first question you ought to ask is, has he had morphine? And, in fact, this patient has had morphine, and that was the cause of his slow breathing.

Dr.—Now the temperature was 98.4 on two occasions and 98.5 on the third day. The question is, what important information do we gain in this case from that fact? What does it enable us tentatively to exclude? S.—It would exclude any acute infection. Dr.—Such as—S.—Peritonitis. Dr.—If it continues normal it is against peritonitis. I would not say that a normal temperature might not occur in general peritonitis. What temperature would you expect? S.—Possibly 102 or 103. Dr.—What would you expect with a typhoid case just after perforation? Suppose a typhoid ulcer had perforated, what temperature would you expect? S.—It would be subnormal. Dr.—General peritonitis has a period of low temperature immediately following the occurrence of its cause. But this is neither subnormal nor raised, and so tends to make us think he hasn't got general peritonitis.

What do you think about the strawberries? You see, his symptoms immediately followed the eating of clam chowder and strawberries. What do you think about that combination of food? S.—If they got down to the region of the hernia they might have constricted it, but since they were vomited, they are probably not of importance. Dr.—If they cause trouble, what kind of trouble would they have been likely to have caused? S.—Strangulation of the hernia. Dr.—Or strangulation of the gut. Do you know any case on record of that? I do not know of any case on record, but I never like to deny anything of that kind because if I tell my students it can't happen, they bring me a reference where it did—I am learning lots from them all the time—but unless you have some reference of that kind in your mind, I think we can say that strawberries have not yet produced that condition. What effect might they have? S.—They might cause acute indigestion. Dr.—What then would probably be the state of his bowels? S.—They would be loose. Dr.—As to the number of movements, were they more or less than before? S.—After the strawberries, less. Dr.—They would probably not lock up his bowels. Apparently this man's bowels used to move; after eating the strawberries they did not move; ordinarily they don't produce that effect, so that it becomes difficult for us to connect them with this event, and yet we can't help noticing that the trouble immediately followed that.

Now, I think it is time to suggest some possible diagnoses. What diagnosis seems one of those possible in this case? S.—Intestinal obstruction.

Dr.—We certainly must consider that as a diagnosis whatever we finally think about it. What suggests it? S.—The constant vomiting. Dr.—That is, whenever a man has constant vomiting, you think of intestinal obstruction. Would you? S.—No, when there is fecal matter or stercoraceous vomiting. Dr.—That is suggestive of obstruction? Yes, what else causes the vomiting of stercoraceous material? [No answer.] Well, there are two common causes, intestinal obstruction and general peritonitis. They are the two common causes, and in respect to that symptom are practically the same thing, for general peritonitis produces obstruction by paralyzing the bowel. General peritonitis has got to be thought of in this case. There is a good deal against it, but we have got to consider it.

Dr.—Beside stercoraceous vomiting, what else is in favor of intestinal obstruction? S.—Hernia. Dr.—Anything else? S.—That there was no passage of feces. Dr.—The fact that his bowels didn't move. First the bowels didn't move, then the stercoraceous vomiting, then the hernia.

Dr.—Now is there any other possible diagnosis? S.—I think appendicitis ought to be considered. Dr.—Would you have to add anything to that diagnosis? Could simple appendicitis produce these symptoms or would you have to say appendicitis plus something else? Would a case of simple stercoraceous vomiting indicate appendicitis? S.—Plus peritonitis. Dr.—Yes; you would have to put that down complicating appendicitis.

Dr.—Is there anything else? S.—I should think obstruction of gallstone. Dr.—What suggested that to your mind? S.—The acuteness of the pain. Dr.—Yes, what else? S.—The tendency to slow pulse and slow respiration. Dr.—Yes, if there were jaundice in the case that would explain the slow pulse, although it is not extraordinarily slow. But what connection is there between gallstone and intestinal obstruction? S.—Gallstone may produce peritonitis, and so bring about obstruction. Dr.—Yes, that is one connection. What other connection between gall stone and intestinal obstruction beside the fact that the gallstone ulcerating through may give peritonitis? Suppose we had 500 small gallstones in gall-bladder, would that produce internal obstruction? S.—I think it might. Dr.—No, it never does. S.—A large stone may perforate directly into the intestine; it is not a very rare case. Dr.—Right. It is a very hard thing to picture to yourself because gallstones seem so small compared to the diameter of the intestine. If the intestinal obstruction is due to gallstone, where would the intestinal obstruction be? S.—In the transverse colon. Dr.—What is the difficulty of its being in the transverse colon? S.—It wouldn't be so likely to ulcerate through into the colon. Dr.—Yes, it might be in such a position that this could happen. S.—The transverse colon is so much larger than the small intestine. Dr.—It is almost unknown to have a gallstone large enough to obstruct it; therefore, if due to gallstone it is probably in the small intestine or duodenum. Now we have reason here to know that this obstruction, if it is obstruction, is not in the small intestine and not in the duodenum. Can any one see what reason we have for believing that it is not in the small intestine? S.—The physical examination gives a mass which seems to correspond to the position of the ascending colon. Dr.—Now any other reason? S.—The vomiting would have came sooner. Dr.—How soon did it come? S.—The second night after he had eaten the strawberries and clam chowder. Dr.—There seemed to be about twenty-four hours between the beginning of the symptoms and the beginning of the vomiting, and that would be a point against the occurrence of obstruction in the small intestine. Can anybody tell me whether the symptoms are apt to be more acute and more severe if obstruction occurs in the small intestine or in the large? Which kind gives the severest symptoms? S.—The small. Dr.—Yes, they are usually worse within a short time than obstructions in the large intestine, and there was another point which we took account of in the beginning. What was that? S.—The pulse and temperature were normal on the third day. Dr.—Yes; that the pulse and temperature were practically normal the third day means obstruction in the large intestine, if anywhere.

Now are there any other possible diagnoses that we want to put down? Does any one think of anything else? S.—Toxic poisoning from the fruit. Dr.—Well, we have discussed that more or less. The difficulty is that toxic poisoning almost always gives us diarrhea. I have never heard of any case of intestinal obstruction following toxic poisoning from food. S.—Are we sure the hernia was reduced? Dr.—I am glad you brought that up. S.—It might have been just reduced in mass. Dr.—Tell me more about that. S.—I think it easily happens in an attempt to reduce hernia that not only the intestine but ring and all are shoved into the abdominal cavity. Dr.—Do you think that happened in this case? S.—Why, I think the temperature remaining at 98.5 and the pulse 68 the third day is rather against it. Dr.—Has any one any other opinion as to the condition of that hernia? What is the likelihood that that went up into the belly without being reduced? Of course, we have no very positive evidence on that point; but it is evident, in the first place, that he had been in the habit of having his hernia come down and putting it back and, although it went back with more difficulty than usual, there was not so much difference as you would expect. In those cases where the whole mass is shoved back without being reduced, great force is used, and here where the patient has reduced it himself you have no reason to suppose anything of that kind.

Dr.—Is there any other diagnosis? My class in Boston insist we shall consider hysteria. I have got in the habit of considering hysteria with everything, because there is no disease almost that it can't simulate; but I must say that I could not produce any evidence that hysteria has ever produced stercoraceous vomiting. The other symptoms could very easily be reduced to hysteria, except that one would have to explain this mass on the right side of the abdomen. What could we do to explain that? S.—It might be a muscle. Dr.—Yes, again and again I find myself deceived by what turns out to be a rectus muscle. What could you do to be sure? S.—You could examine it in a warm bath. Dr.—Yes, that would be much better than examining it under anesthesia. Does any one know any other test for diagnosing a rectus muscle from a true abdominal tumor? [No answer.] You have the patient lie on a table and raise his head while your hand is on the supposed mass and if the mass is muscle it thickens up instantly. That is pretty good as a preliminary test; any tumor which becomes more prominent when the patient raises his head is likely to be rectus muscle. What else might it be, if not rectus muscle? S.—A floating kidney. S.—A phantom tumor. S.—The presence of gas. Dr.—Yes, but of what organ or what viscus? Sometimes in connection with a dilated colon one gets very large "phantom" tumors which appear as the gas is set free; it might be a mass of feces, although they almost always have a stricture ahead of them if they accumulate. But it does not seem to me on the whole, necessary to consider hysteria.

Now, our time is short and though there are many other points to discuss I shall have to stop and take a vote between these two diagnoses (on blackboard): 1. Intestinal obstruction. 2. General peritonitis. How many vote for the first? How many for the second? It seems to be unanimous for the first, and I agree with the majority. And if it is intestinal obstruction, where is it and what is it due to?

We have already discussed a little the question where it is. It seems much more likely that it is in the large intestines because the symptoms are not so acute, and from the position of this mass which seems to correspond to the ascending colon. Now whereabouts is it? S.—In the ascending colon. Dr.—This mass may be what? S.—Feces. Dr.—Yes; if it were a mass of feces then the obstruction would be where? Either in the transverse or ascending colon or where else? S.—The sigmoid. S.—The hepatic flexure. Dr.—Of course the whole mass might be malignant disease, but it is a pretty large mass for malignant disease. If it is in the large intestine and in the neighborhood of the hepatic flexure, what is it most likely to be due to? S.—It might be intussusception. Dr.—What is the common age for that? S.—It is found in children. Dr.—This, then, is not a common age for it? S.—The obstruction may be due to previous operation. Dr.—That is a common cause, but we have not had any evidence that he has had a previous operation. Is there any other possibility of a cause for this obstruction? Now, taking just the fact of his age, what is the common cause of intestinal obstruction at his age? S.—It might be due to stricture. Dr.—Of what nature? S.—Twist. Dr.—Twist is a rare cause. What is more common at his age? S.—Ulcer. Dr.—That is rather rare. S.—Hernia. Dr.—Yes. S.—He may have a neoplasm. Dr.—Yes, that is about five times as common as any of the rest of those; at this age, sixty-three, your snap diagnosis should be malignant disease. Now, if it were malignant disease, how could it have produced acute obstruction in this way? That is the difficulty that occurs to you. Malignant disease is a slow-growing process; this apparently came on acutely? Is there any way to reconcile these facts? S.—He may have been constipated for some time and then there may have been acute spasm. Dr.—Well, an element of spasm might have come in by reason of excessive peristalsis; but what would suggest a possible cause for increased peristalsis? S.—The strawberries. Dr.—Yes.

We have wanted all along to get the connection that the strawberries and clam chowder have with this case, and you suggest a possible way of associating them. Malignant disease may exist for a long time in the large intestine and give no sign of its presence; and then some acute cause like this may set up peristalsis and tighten up the stricture so as to cause this acute obstruction. Again and again I have seen malignant disease of the intestine come on just as this has because some particular cause like the strawberries and clam chowder made the stricture shut down. Now, what further examination would help you at all in determining the nature of this obstruction? Is there anything else you could do so as to be any surer as to what this thing is? S.—If you could get a movement of the bowels, you would have a chance to examine the stools. Dr.—How would you advise us to get that movement? S.—You might give a cathartic. Dr.—What cathartic would you give? S.—I would not give any. Dr.—That's right, but how would you get it? S.—You might try hot applications. Dr.—Enemas have already been tried. S.—You might try some more. Dr.—Would you suggest any different kind of enema? Could you think of any other kind that could be given? S.—You might give a high enema, with a stimulant, such as turpentine. Dr.—It is occasionally worth while to try the high enema with oil, but it seems to me as if they had given a pretty fair trial to enemata here. They got in five and one-half quarts. How high would that go? S.—I should think that would go through the descending and transverse colon. Dr.—Yes, one can tell by that fact that the obstruction is not low down. Would blood examination help you any? S.—I think so. Dr.—What would you do? S.—Look for a leucocytosis. Dr.—How would that help you? S.—In a case of malignant disease anemia would be present. Dr.—I don't think it would be likely to help you; malignant disease might have produced an anemia, but his previous history was stated as excellent, so he would not be likely to be anemic. Would the urine examination help you any? S.—Indican. Dr.—What would that tell? S.—It would show that there was intestinal obstruction. Dr.—What else beside intestinal obstruction will give you an increased amount of indican? Aren't there a good many other conditions where indican is increased? S.—It might be due to constipation. Dr.—What kind of intestinal obstruction increases indican? S.—When you have any putrefactive changes. Dr.—And if intestinal obstruction, what kind would be most likely? S.—It would be far down in the intestine. Dr.—What do you think about that? You think it is usually obstruction high up? How many think it is low down? How many high up? The majority seems to be for low down. That is wrong. Usually it is high up; obstruction high up is much more likely to give rise to an increase of indican than obstruction low down; it is due, also, to many causes where there is no obstruction at all. I don't think the estimation of indican would be likely to help us much in this case. Some writers have laid stress on it, but don't think the majority have found it of any use in diagnosis.

Practically one can't go further in this case without laparotomy. It was done in this case, and a very small tight cancerous stricture was found, not much bigger than a signet ring, in the region of the hepatic fixture. The hernia was practically reduced and did not count.

Given these facts, what is the prognosis? S.—That would depend upon whether there were secondaries or not. Dr.—Where would you look for them? S.—In the retro-peritoneal region. Dr.—Certainly. What else would it depend on? S.—The extent of the growth. Dr.—Anything else? S.—The condition of the patient. Dr.—What else. S.—The length of time. Dr.—Yes, every day makes the prognosis worse. But there is another element—who does the operation. That is one of the personal elements we can not eliminate in a case like this. If it is done by a man of experience it is of greater value than if done by a greenhorn. We have recently published in Boston some stomach statistics of operations gathered from one hundred and fifty cases, and one is appalled at the mortality. The average was about four cases to an operator. Everybody was getting his experience on those cases, and consequently there was a very high mortality. On the next one hundred and fifty cases in Boston, it won't be so high. So the things to be considered are the general condition of the patient, the length of time, the presence or absence of metastases, the man who does the operation, and, of course, the after-treatment and nursing. We have nothing especial to say about the treatment in this case other than surgical. The medical treatment of intestinal obstruction is not a subject that one is inclined to dwell on with pride.—*Detroit Medical Journal.*

---

### CHRONIC CONSTIPATION: A RATIONAL TREATMENT.

Dr. Dwight H. Murray, of Syracuse, N. Y., in a paper on this subject read before the Medical Society of the County of New York, referred to the pathological changes that occur in the rectum, particularly the anal canal, and also to proctitis, sigmoiditis, and colitis. As a rule, the pathological changes that are associated with chronic constipation are to be found below the entrance to the sigmoid. His plan of treatment of chronic constipation had not been used by others, and he said it gave him much pleasure to be able to present it to the profession.

In taking the patient's history, the most careful routine should be followed, and certain symptoms should be elicited that would aid in classifying the cases. A thorough examination was necessary; if patients declined to allow such an examination, they should be refused treatment. The treatment was given every fourth day, until the patient had daily normal stools; after that time the length between treatments should be increased, until the patient could get along on his own resources.

The treatment occupied about one hour. The patient was placed in Sims' position, and an electrode with a perforated soft rubber shield was passed into the sigmoid. This was connected with the positive pole of a battery, and also with an irrigator containing decinormal salt solution, held three and a half feet above the patient; the flow was readily controlled by a stop-cock. Before treatment is begun, the mucosa should be thoroughly cleansed. The effect of the treatment seemed to be soothing and quieting to the mucous membrane. As a result of treatment the patients had a better power of expulsion than was possible before the treatment was begun. The temperature of the fluid used should be 100° F., and from five to twenty milliamperes should be used. When the first part of the treatment was finished, the skin would be found to be reddened. No electricity reached the part except through the saline solution. The electricity was applied about ten or fifteen minutes, and from thirty-two to thirty-six ounces of fluid flowed through into the sigmoid. Then the patient was instructed to go to the toilet. Later he returned to the table for the second part of the treatment, when a special tube was utilized to insert into the colon bismuth subnitrate, iodoform, and oil. This was followed by the use of a solution of ichthyol or hydrastis. The patients were kept on the table with the hips elevated for ten minutes. When the treatment was begun, they were instructed to discontinue the use of all laxatives, to go to the toilet at certain hours every day, and never to strain. If the bowels at first failed to move, then an enema of plain water and oil was given. The patients were allowed a liberal diet, and were told to drink six glasses of water daily.

The results of this treatment were that daily normal movements were produced. Nearly seventy-five per cent. were cured, while almost all of the remaining twenty-five per cent. were improved. Four and a half years was the longest time required in the treatment. Dr. Murray closed his paper by stating that the three greatest causes of constipation were ignorance, carelessness, and laziness, and he believed that a great many people to-day in the insane hospitals were brought there by the results of chronic constipation.—*Medical Record.*

---

### Provide Another Outlet.

For some years the profession has felt strongly that there are too many medical schools and too many ill-equipped physicians. We all know that many schools are very "lame." We speak of them freely among ourselves, but few of us mention them in print for fear of hurting the feelings of some of our friends. The task of dealing with the problem has been left to the Council on Medical Education of the American Medical Association.

In the meantime we are all seeking remedies for this ill condition that is so detrimental to the welfare of the profession. Ohio suggested a remedy when it took the great step forward of passing a State law providing a minimum educational requirement for entrance to medical schools and gave its enforcement into the hands of the impartial public school system. New York has long had a similar regulation controlling entrance to medical colleges. Kentucky, on July 1, 1905, enters the same company. This method is excellent, in that it excludes the unfit at the beginning. The work, then, is well begun, and other States must soon fall in line or suffer the uncomfortable consequences.

But why have we too many medical schools? Can we not also tackle the problem simultaneously from the top? And, lastly, can we not provide some recompense to those who must lose teaching positions as schools are closed? The first question is not difficult to answer. As we enter the profession all of us desire fame and reputation, as well as full pocketbooks. Fame comes slowly to him who confines himself to private practice. A hospital appointment and a professorship constitute alluring and promising short-cuts. Secondly, can we find a means to attain reputation that will attract us away from the professorships; and thirdly, that will furnish continued opportunity to those crowded out of places by the diminution in the number of schools? It is believed that these two questions can be answered in the affirmative.

In a large part of the United States the existing profession is sorely in need of postgraduate instruction to enable it to fulfill its whole duty to itself and to the community. One of the aims of our perfected organization was the facilitating of postgraduate study and instruction. Why not, then, arrange to combine these mutually satisfying needs? Let the schools and teachers soon to be without students set themselves to the splendid task of improving the vast number of physicians who necessarily must live far from clinics and laboratories. Why not provide for systematic and semi-compulsory postgraduate training of the members of our organization? Having thus combined these two needs it will be found that fame is to be attained in teaching physicians just as formerly in instructing undergraduates. He who wants to teach will have just as good a chance as he ever had. The number of members in an overcrowded profession will be steadily reduced. The educational qualifications of the present generation of physicians will be materially improved. The only drawbacks lie in the friction incident to the initiation of the process. If the State medical associations take hold of the matter in proper spirit, the difficulties will soon fade. The proposal is far from being chimerical, and it is confidently believed that the next few years will somewhere witness a trial of this method of disposing of the most vexed problem that demands solution at the hands of the organized profession.—*Journal A. M. A.*

---

### ARTIFICIAL SUBCUTANEOUS NOURISHMENT IN PRACTICAL SURGERY.

BY DR. FRIEDRICH,

(*Arch. f. klin. Chir.*, LXXIII.; ref., *Fortschritte der Medizin*, January 10, 1905, p. 59.)

The author started with the idea that, in cases of abdominal surgery in which the natural nutrition via the digestive organs is impaired, or almost entirely suspended, a temporary, though limited substitute for this defect could be found in artificial nourishment applied directly to the tissues. For many years he experimented in this direction, and first endeavored to meet the demands of the body for calories by subcutaneous infusion of grape sugar solutions isotonic with the blood. For this purpose he found that solutions containing 2 pro mille of NaCl and from 30 to 35 pro mille of chemically pure grape sugar (Merck) were best. Of these solutions—which had previously been boiled for ten minutes—he injected at blood temperature from one to two liters daily under aseptic precautions. At the same time from 20 to 100 grams of sterilized oil were subcutaneously injected.

An albuminous substance suitable for subcutaneous absorption was finally secured in a pure, permanent albumose-free pepton—so-called pepsin-fibrin-pepton. This is a simple, non-coagulable albumin body which can be sterilized for ten to thirty minutes in the steam sterilizer without decomposition, and is taken up and borne by the body without any disturbance in doses up to a maximum of 20 grams=100 calories.

The following method of administration proved most practical: In the forenoon 1000 cc. water containing 2.0 gms. NaCl, 35 gms. grape sugar, and 15 gms. pepton. Towards evening repetition of the infusion with the same amount of water, common salt and sugar, but only 5 to 10 gms. of pepton. In the interval a single injection of from 20 to 100 gms. of olive oil.

While this substitution of albumin does not prevent further destruction of the body albumin, it markedly diminishes the loss, so that in cases of peritonitis, gastric and high-seated intestinal perforation, it is possible to sustain the patient for a period of from ten to fourteen days on an exclusively subcutaneous supply of nutriment.

---

### FAT EMBOLISM OF THE LUNG AFTER FRACTURES.

F. Gregory Connell, Salida, Colo. (*Journal A. M. A.*, February 25), remarks on the rarity of American literature on this subject. He reports two cases. In practically all fractures, he says, there is more or less fat embolism, according as there is comminution, rough handling, etc. It may also follow orthopedic operations, surgical procedures, inflammations, fatty liver, etc., and the most striking feature is the preliminary period of euphoria, while the most important clinical symptom is the presence of fat in the urine or sputum. Later respiratory and cerebral symptoms may appear at any time up to the fifteenth day. The condition is frequently overlooked or confused with shock, septicemia, pulmonary embolism, effects of anesthetics, etc. As we do not know its frequency its prognosis is uncertain. It probably occurs in a mild form after a large proportion of fractures and is unrecognized. The only treatment that is practically available is judicious heart stimulation.

